# Skull Base Osteomyelitis Due to Staphylococcus epidermidis in the Absence of Indwelling Medical Devices Presenting With Bilateral Internal Carotid Artery Thrombosis

**DOI:** 10.7759/cureus.66563

**Published:** 2024-08-10

**Authors:** Allahdad Khan, Abdul Ahad Riaz, Shahroze Ahmad, Ahmad Shabbir, Abdul Sattar Anjum

**Affiliations:** 1 Department of Medicine, Nishtar Medical University, Multan, PAK; 2 Department of Radiology, Shaukat Khanum Memorial Cancer Hospital and Research Centre, Lahore, PAK; 3 Department of Radiology, Nishtar Medical University, Multan, PAK

**Keywords:** neurological signs, staphylococcus epidermidis, infarct, thrombosis, internal carotid artery, skull base osteomyelitis

## Abstract

Skull base osteomyelitis (SBO) is a severe and uncommon infection that typically affects the skull base and may arise from undiagnosed otogenic or sinonasal infection. This case describes a rare presentation of SBO, accompanied by thrombosis of the bilateral internal carotid artery with neurological deficits in a resource-limited environment, illustrating diagnostic and management dilemmas. A male patient aged 40 years with poorly controlled type 2 diabetes presented with sudden onset loss of consciousness and worsening right-sided weakness. MRI studies revealed SBO with cerebral involvement with thrombosis in major cerebral arteries and multiple brain infarcts. After receiving broad-spectrum antibiotics and supportive care shortly after admission, the patient developed septic shock and died two days after admission. The fast course of the disease in this case shows how severe SBO and its complications may be, calling for early diagnosis and intensive management of SBO, especially in diabetic patients. The fact that Staphylococcus epidermidis was established as a causative agent of disease in the absence of artificial heart valves or joints, it is becoming clear that there is a need to increase awareness of such rare pathogens, and probably new strategies for handling such infections should be developed. Additional research is required to elucidate the precise role of the pathogen and refine treatment approaches, especially for low-resource healthcare systems.

## Introduction

Osteomyelitis is an inflammatory bone disease caused by an infection [1], with skull base osteomyelitis (SBO) specifically involving the temporal, sphenoid, or occipital bones. This condition often results from untreated otogenic or sinonasal infections in elderly diabetic or immunocompromised patients, although it can also occur without prior sinonasal or otogenic infection involvement [2]. The prevalence of SBO accounts for approximately 1.5% of all osteomyelitis cases [3].

Bone infections such as osteomyelitis can arise through various routes: hematogenous spread from a distant infection site, direct extension from nearby tissues and joints, or direct inoculation during trauma or surgery [4]. Diabetes is a significant risk factor, particularly influencing the duration and severity of otogenic SBO compared to non-otogenic cases. Additional risk factors include recent head or neck surgery and elevated creatinine levels. The type of infection also varies; bacterial infections predominantly occur in otogenic cases, while non-otogenic cases may involve both bacterial and fungal pathogens [5].

According to a population-based study from 1969 to 2009, the annual incidence of osteomyelitis as a whole is 21.8 cases per 100,000 person-years, with higher rates observed in men and increasing prevalence with age [6]. The overall mortality rate for osteomyelitis stands at 36.7%, rising to 45% in the presence of cranial nerve palsies [7].

This case report documents a rare instance of bilateral thrombosis of the internal carotid artery resulting from SBO, leading to significant neurological sequelae. Notably, the causative organism, Staphylococcus epidermidis, has rarely been reported in the literature especially in patients without artificial heart valves or joints to our knowledge, making it impossible to determine the global or Pakistani incidence of this specific combination. We aim to document a rare condition with its diagnostic and therapeutic challenges, especially in resource-limited settings.

## Case presentation

A 40-year-old man presented in the emergency department with sudden onset loss of consciousness and worsening right-sided weakness. The patient had type 2 diabetes poorly controlled for five years now and has been bedridden for the last five months due to severe weakness. His family also reported that he had been having continuous headaches for two weeks prior to the current episode and possibly had a transient ischemic attack (TIA) previously indicated by his medical records. The patient has never smoked any cigarettes and never used any alcohol or illicit drugs in her lifetime.

At first glance, he looked very sick. His vitals were as follows: blood pressure (180/100 mmHg), pulse rate (106 beats/min), respiratory rate (25 breaths/min), and temperature (98.8 °F). His HbA1c levels were at 8.1%, and random blood sugar was 320 mg/dL. A neurological examination revealed decreased consciousness (Glasgow Coma Scale (GCS): 11/15), increased muscle tone in the right upper and lower extremities, and significantly reduced strength on the right side. His plantar reflex was abnormal on the right side as well. Over the next two days, his level of consciousness further declined (GCS: 8/15), he developed a fever (100 °F), and his breathing became more labored. Repeat neurological exams showed complete paralysis (motor strength: 0/5) on his right side. A complete blood profile revealed elevated leukocytes, predominantly neutrophils.

**Figure 1:**
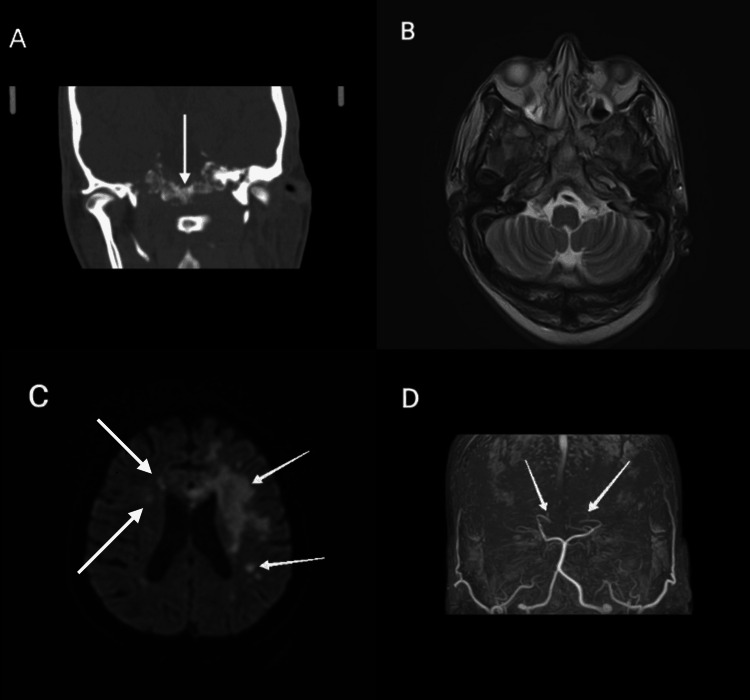
A (coronal CT head bone window) and B (MRI brain axial T2-weighted image) showing osteopenia and altered signals along the base of the skull, i.e., body and greater wings of the sphenoid bone. C (MRI brain axial DWI) showing significant diffusion restriction in the left fronto-parietal, medial temporal, capsuloganglionic regions and the genu of the corpus callosum suggestive of acute infarcts, in addition to multiple small right-sided acute infarcts. D (MR cerebral angiogram) showing non-visualization of bilateral ICAs and anterior circulation, suggestive of thrombosis

A CT brain along with an MRI brain was done, which revealed extensive sinus and ear infections complicated by osteomyelitis of the skull base, resulting in thrombosis of the petrous and cavernous parts of bilateral internal carotid arteries (ICAs), and multiple bilateral infarcts involving cortical and subcortical regions, including the frontal and parietal lobes, bilateral rectus gyri, left medial temporal lobe, genu of the corpus callosum, corona radiata, centrum semiovale, and bilateral capsulo-ganglionic regions (Figure 1).

A neurology consult suspected a combination of uncontrolled diabetes, a previous possible stroke, and a severe CNS infection behind the patient’s deteriorating clinical status. A lumbar puncture was done to analyze cerebrospinal fluid, which showed the presence of S. epidermidis, in addition to elevated white blood cells, primarily neutrophils, elevated protein levels, and decreased glucose levels. The patient was started on a large array of medications, including antibiotics, analgesics, anticonvulsants, anticoagulants, proton pump inhibitors, mannitol, sedatives, and insulin. Supportive measures included an air mattress and chest physiotherapy. Despite these measures, the patient deteriorated further and went into septic shock (indicated by hypoxemia, acidosis, elevated lactate levels, and tachycardia) and died after two days.

## Discussion

In SBO cases, bacterial pathogens are predominantly involved, most commonly Pseudomonas aeruginosa (42%) [8]. Other bacterial agents include Staphylococcus aureus, Escherichia coli, Citrobacter koseri, Morganella morganii, and coagulase-negative Staphylococcus [9]. Case studies of SBO, particularly those involving S. epidermidis, are typically found in individual or smaller-scale investigations due to their rarity in broader epidemiological studies.

Complications such as cerebral venous thrombosis and arterial infarcts frequently arise in SBO cases. The development of cerebral venous thrombosis occurs through the dissemination of infection via diploic veins, resulting in thrombophlebitis of the sagittal sinus. Additionally, SBO can spread to neighboring tissues and infiltrate the adventitia of the ICA, potentially leading to the formation of an arterial pseudoaneurysm characterized by a false lumen [6]. This leads to the formation of watershed infarcts in the brain, resulting in necrosis.

Due to the involvement of temporal, parietal, and retro-orbital areas, severe and profound otalgia presents in a patient with SBO with spiking fever, aural fullness, and foul purulent otorrhea. In some patients, SBO can present atypically with persistent headaches, even without localized ear or sinus infections, making diagnosis difficult. This infection may result in early cranial neuropathies, such as abducens nerve palsy [7].

Identifying the precise diagnosis poses the initial hurdle in managing SBO. No single imaging technique can fully capture the scope of this condition, and it is challenging to differentiate active inflammation from resolving issues using conventional imaging methods. Nuclear medicine functional imaging, either individually or paired with high-resolution anatomical imaging such as single-photon emission computed tomography (SPECT) or PET with CT or MRI, is suggested but not commonly accessible [10]. The gold standard test for osteomyelitis is bone biopsy with histopathologic examinations and tissue culture [5]. The management of SBO consists of both surgical intervention and prolonged antibiotic therapy. Early intervention leads to fewer complications and better prognosis but still will take several months for complete resolution. Effective management of SBO entails addressing underlying comorbidities, including implementing rigorous glycemic control for diabetes and enhancing immune function in immunocompromised individuals [5,11].

Given the intricate nature of SBO, it is likely that the duration of treatment will require individualized adjustment according to each case. The average length of intravenous antimicrobial therapy (IV AMT) was approximately 6.8 weeks, followed by an average total duration of AMT of about 15.7 weeks [12]. The prognosis for SBO is grave, characterized by the potential for recurrence, leading to subsequent hospitalizations and a high mortality rate [5].

Several factors influence the prognosis of SBO. The type of osteomyelitis itself plays a role, with central SBO (CSBO) patients experiencing significantly longer durations of symptoms before diagnosis compared to those with lateral SBO (LSBO). Additionally, CSBO cases often require more biopsies to confirm the diagnosis. Furthermore, the presence of fungal cultures in a patient's microbial profile significantly impacts the duration and complexity of their AMT, suggesting a more challenging management course [12].

## Conclusions

SBO is a rare and severe infection primarily affecting the skull base. It often arises from untreated otogenic or sinonasal infections in elderly diabetic or immunocompromised patients, although it can also occur without prior infection. Our case involved a unique instance of SBO complicated by thrombosis of the bilateral ICA, leading to neurological symptoms. The identification of S. epidermidis as the causative agent highlights the importance of considering this pathogen in diagnostic evaluations and potentially revising treatment protocols to address such rare infections more effectively. Further research is needed to better understand the role of this pathogen and to optimize therapeutic strategies, especially in resource-limited healthcare settings.

## References

[1] Jha Y, Chaudhary K (2022). Diagnosis and treatment modalities for osteomyelitis. Cureus.

[2] Álvarez Jáñez F, Barriga LQ, Iñigo TR, Roldán Lora F (2021). Diagnosis of skull base osteomyelitis. Radiographics.

[3] Sayhan MB, Kavalci C, Sogüt O, Sezenler E (2011). Skull base osteomyelitis in the emergency department: a case report. Emerg Med Int.

[4] Lew DP, Waldvogel FA (1997). Osteomyelitis. N Engl J Med.

[5] Das S, Iyadurai R, Gunasekaran K (2019). Clinical characteristics and complications of skull base osteomyelitis: a 12-year study in a teaching hospital in South India. J Family Med Prim Care.

[6] Kremers HM, Nwojo ME, Ransom JE, Wood-Wentz CM, Melton LJ 3rd, Huddleston PM 3rd (2015). Trends in the epidemiology of osteomyelitis: a population-based study, 1969 to 2009. J Bone Joint Surg Am.

[7] Auinger AB, Dahm V, Stanisz I, Schwarz-Nemec U, Arnoldner C (2021). The challenging diagnosis and follow-up of skull base osteomyelitis in clinical practice. Eur Arch Otorhinolaryngol.

[8] Reshma SA, Noushad M (2022). Skull base osteomyelitis: not just Pseudomonas. Am J Otolaryngol Head Neck Surg.

[9] Vlastos IM, Helmis G, Athanasopoulos I, Houlakis M (2010). Acute mastoiditis complicated with bezold abscess, sigmoid sinus thrombosis and occipital osteomyelitis in a child. Eur Rev Med Pharmacol Sci.

[10] Sreepada GS, Kwartler JA (2003). Skull base osteomyelitis secondary to malignant otitis externa. Curr Opin Otolaryngol Head Neck Surg.

[11] Khan MA, Quadri SA, Kazmi AS (2018). A comprehensive review of skull base osteomyelitis: diagnostic and therapeutic challenges among various presentations. Asian J Neurosurg.

[12] Tan D, Trent MS, Wang E (2024). Duration and prognostic factors of therapy in skull base osteomyelitis: a bi-institutional analysis. J Neurol Surg B Skull Base.

